# Spectrum of Rare and Common Genetic Variants in Arrhythmogenic Cardiomyopathy Patients

**DOI:** 10.3390/biom12081043

**Published:** 2022-07-28

**Authors:** Melania Lippi, Mattia Chiesa, Ciro Ascione, Matteo Pedrazzini, Saima Mushtaq, Davide Rovina, Daniela Riggio, Anna Maria Di Blasio, Maria Luisa Biondi, Giulio Pompilio, Gualtiero I. Colombo, Michela Casella, Valeria Novelli, Elena Sommariva

**Affiliations:** 1 Unit of Vascular Biology and Regenerative Medicine, Centro Cardiologico Monzino IRCCS, 20138 Milan, Italy; mlippi@ccfm.it (M.L.); drovina@ccfm.it (D.R.); gpompilio@ccfm.it (G.P.); 2 Department of Medicine and Surgery, Università degli Studi di Milano Bicocca, 20126 Milan, Italy; 3 Bioinformatics and Artificial Intelligence Facility, Centro Cardiologico Monzino IRCCS, 20138 Milan, Italy; mchiesa@ccfm.it; 4 Department of Electronics, Information and Biomedical Engineering, Politecnico di Milano, 20133 Milan, Italy; 5 Heart Rhythm Center, Centro Cardiologico Monzino IRCCS, 20138 Milan, Italy; ciroascione92@gmail.com; 6 Department of Clinical Sciences and Community Health, Università degli Studi di Milano, 20122 Milan, Italy; 7 Laboratory of Molecular Genetics, Istituto Auxologico Italiano IRCCS, 20145 Milan, Italy; m.pedrazzini@auxologico.it (M.P.); a.diblasio@auxologico.it (A.M.D.B.); 8 Cardiovascular Imaging Department, Centro Cardiologico Monzino IRCCS, 20138 Milan, Italy; smushtaq@ccfm.it; 9 Operative Unit of Clinical Chemistry and Hematology, ASST Santi Paolo e Carlo, 20142 Milan, Italy; daniela.riggio@asst-santipaolocarlo.it; 10 Unit of Laboratory Medicine, Centro Cardiologico Monzino IRCCS, 20138 Milan, Italy; mbiondi@ccfm.it; 11 Department of Biomedical, Surgical and Dental Sciences, Università degli Studi di Milano, 20122 Milan, Italy; 12 Unit of Immunology and Functional Genomics, Centro Cardiologico Monzino IRCCS, 20138 Milan, Italy; gcolombo@ccfm.it (G.I.C.); vnovelli@ccfm.it (V.N.); 13 Department of Odontostomatologic and Specialized Clinical Sciences, Università Politecnica delle Marche, 60121 Ancona, Italy; m.casella@univpm.it

**Keywords:** arrhythmogenic cardiomyopathy, cardiovascular genetics, genotype-phenotype correlation, desmosomal genes, rare variants, common variants

## Abstract

Arrhythmogenic cardiomyopathy (ACM) is a rare inherited disorder, whose genetic cause is elusive in about 50–70% of cases. ACM presents a variable disease course which could be influenced by genetics. We performed next-generation sequencing on a panel of 174 genes associated with inherited cardiovascular diseases on 82 ACM probands (i) to describe and classify the pathogenicity of rare variants according to the American College of Medical Genetics and Genomics both for ACM-associated genes and for genes linked to other cardiovascular genetic conditions; (ii) to assess, for the first time, the impact of common variants on the ACM clinical disease severity by genotype-phenotype correlation and survival analysis. We identified 15 (likely) pathogenic variants and 66 variants of uncertain significance in ACM-genes and 4 high-impact variants in genes never associated with ACM (*ABCC9*, *APOB*, *DPP6*, *MIB1*), which deserve future consideration. In addition, we found 69 significant genotype-phenotype associations between common variants and clinical parameters. Arrhythmia-associated polymorphisms resulted in an increased risk of arrhythmic events during patients’ follow-up. The description of the genetic framework of our population and the observed genotype-phenotype correlation constitutes the starting point to address the current lack of knowledge in the genetics of ACM.

## 1. Introduction

Arrhythmogenic cardiomyopathy (ACM) is a rare cardiac genetic disorder, presenting incomplete penetrance and variable expressivity. ACM mainly affects young people, especially males, despite autosomal inheritance [1]. ACM ventricular myocardium is characterized by wide cardiomyocyte death, inflammation and fibro-adipose tissue replacement, with the consequent electrical instability and mechanical impairment. The expressivity of the disease is highly variable, and patients’ symptoms may range from the onset of premature ventricular contractions (PVC) to malignant ventricular arrhythmias and sudden cardiac death (SCD). In addition, contractile dysfunctions can occur and progress to the point of heart failure (HF) [1].

ACM patients show predominantly right ventricle (RV) involvement, according to the 2010 diagnostic International Task Force Criteria, which are based on several parameters, comprising functional and structural abnormalities of RV, histological characterization of the wall, electrocardiographic depolarization and repolarization alterations, ventricular arrhythmias, family history and genetic background [2]. Nevertheless, lately, left and biventricular forms of ACM have been recognized [3,4], and the Padua group proposed additional criteria for the diagnosis of ACM which include left ventricle (LV) dysfunctions, ranging from morpho-functional and tissue abnormalities to electrical alterations specific for LV [5]. 

The 30–50% of cases carry at least one disease-causing mutation [6] in genes encoding for desmosomal proteins, including plakophilin-2 (*PKP2*) (the most frequently involved), plakoglobin (*JUP*), desmoplakin (*DSP*), desmoglein-2 (*DSG2*) and desmocollin-2 (*DSC2*). Less commonly, non-desmosomal genes such as transmembrane protein 43 (*TMEM43*), desmin (*DES*) and phospholamban (*PLN*) are associated with ACM either with definitive or with moderate evidence [7]. The inheritance is typically autosomal dominant; nevertheless, in some cases, homozygous pathogenic variants have been reported associated with syndromic forms, such as Naxos disease or Carvajal syndrome [8], and with a cardiac-restricted phenotype [9]. 

Whereas many ACM patients do not carry causative variants in known associated genes, both in familial and nonfamilial forms [10], others harbor compound or digenic heterozygous mutations [11,12]. In some cases, multiple variants have been found to be associated with disease severity or specific subclinical characteristics [13,14]. Other genotype-phenotype studies have been conducted investigating the correlation between the single causative mutation and the clinical features of the carriers [4,15,16,17,18], while limited studies have been conducted on the contribution of frequent genetic variants (polymorphisms) as phenotypic modulators [19].

The first goal of the study was to describe the genetic framework of a single-center ACM cohort, considering both genes previously investigated for ACM and genes associated with other cardiovascular diseases, with the future perspective to improve ACM genotyping. The second aim was to correlate common variants identified in ACM patients with clinical parameters related to the phenotype, thus evaluating their impact on the clinical disease severity.

## 2. Materials and Methods

### 2.1. Ethics Statement

This study complies with the WMA Declaration of Helsinki and the Department of Health and Human Services Belmont Report. An informed consent form, approved by Istituto Europeo di Oncologia-Centro Cardiologico Monzino Ethics Committee, was signed by all participants.

### 2.2. Study Population

The studied population includes 82 consecutive unrelated ACM probands fulfilling the 2010 International Task Force Criteria (TCF) [2] or ACM diagnostic Padua Criteria [5], recruited at Centro Cardiologico Monzino IRCCS from 2014 to 2020. An exhaustive clinical characterization was conducted for each patient, including, when available: family history (sudden cardiac death, ACM, other cardiomyopathies), lifestyle (sport, smoke), comorbidities (obesity, hypertension, diabetes, coronary artery diseases, autoimmune or inflammatory pathologies), pharmacological therapy (antiarrhythmics, ACE inhibitors, angiotensin receptor blockers), symptomatology (arrhythmic events, lipothymia/syncope), results of routine or invasive diagnostic investigations (blood tests, 24 h Holter, 12-lead electrocardiogram (ECG), transthoracic echocardiogram (ECHO), cardiac magnetic resonance (CMR), biopsy sample, electrophysiological study (EPS) and electro-anatomical mapping (EAM)), medical interventions (trans-catheter ablation, implantable cardioverter-defibrillator (ICD) implant). The clinical variables were considered “primary” when describing proband characteristics (e.g., number of PVCs in 24 h, ejection fraction values) and “secondary” if reporting clinical decisions based on the patient primary clinical overview (e.g., ICD implantation, antiarrhythmic therapy).

### 2.3. DNA Sequencing and Primary Bioinformatic Analysis

Patients’ DNA was extracted from blood. Next-generation sequencing (NGS) was performed using the MiSeq platform with the 150 bp paired-end protocol, (Illumina, San Diego, CA, USA) with the TruSight™ Cardio Sequencing Kit. 

All the 174 genes related to inherited cardiac diseases included in the panel were analyzed (Supplementary Table S1). Analysis was performed using the MiSeq Reporter software (Illumina, San Diego, CA, USA, v.2.6), adopting hg19/GRCh37 as genome reference.

### 2.4. Variant Filtering and Classification

All variants have been annotated by Ensembl Variant Effect Predictor (VEP). Only variants with a read coverage above the 30× were considered for further analysis.

Allele frequency was obtained from Genome Aggregation Database (gnomAD), when available. A threshold of allele frequency below 6.7 × 10^−5^ was used to discriminate between rare vs. common variants [20]. Supplementary Figure S1 describes the analysis workflow. Only coding sequence and splicing variants have been considered for rare variant interpretation, which was performed using the American College of Medical Genetics and Genomics/Association for Molecular Pathology (ACMG/AMP) guidelines [21]. Splicing variants were evaluated with SpliceAI. Common variants were used to perform a genotype-phenotype correlation analysis. 

### 2.5. Statistical Analyses

Genotype-phenotype correlation study was performed using common variants and clinical variables of the patients. Fisher’s exact test or Pearson’s correlation were performed when the variables were categorical or continuous, respectively. A *p*-value less than 0.005 was considered significant. 

For specific variants, which were associated with arrhythmic events and major arrhythmic events, we performed an event-free survival analysis for the different genotypes, by Cox regression models, shown as Kaplan-Meier curves. The log-rank test has been used to calculate *p*-values. The hazard ratios and the corresponding 95% confidence intervals were also provided.

Categorical data were presented as absolute counts and percentages, while continuous data were presented as median values with interquartile range. Follow-up time was reported as mean ± standard error. Analyses and plot were performed by ‘R’.

## 3. Results

### 3.1. Clinical Characteristics of the Cohort

A total of 82 patients were enrolled in the study. The clinical characteristics of the patients are summarized in Table 1. Male sex was prevalent (80.5%). In total, 12 patients (14.6%) presented a family history of sudden cardiac death, and 17 (20.7%) had a family history of cardiomyopathies. 

The age of onset was 40.5 (±1.8) years old. As regards comorbidities, 17 (20.7%) patients were affected by hypertension, 2 (2.4%) by diabetes, 4 (4.9%) by obesity, 8 (9.8%) by coronary artery disease, and 8 (9.8%) by autoimmune disease. As concerns lifestyle, 16 (19.5%) probands were smokers and 31 (37.8%) practiced sports, 9 (11%) of whom endurance type.

Patients underwent most routine diagnostic tests, including 12-lead ECG, 24 h ECG Holter monitoring, cardiac ECHO and CMR. In addition, most patients undertook invasive evaluations, such as EPS, EAM and endomyocardial biopsy collection. 

Thirty-nine (47.56%) subjects presented premature ventricular contractions and 50 (61%) experienced at least one non-sustained ventricular tachycardia (NSVT) or sustained ventricular tachycardia (SVT) or ventricular fibrillation (VF) episode; among these 50 subjects, 35 had major arrhythmias (SVT or VF) and 21 experienced further arrhythmic events in the follow-up (mean follow-up time: 8.6 ± 0.7 years); 15 patients (18.3%) also displayed atrial arrhythmias. ECG alterations were found in 67 (78.0%) probands, of whom 4 displayed epsilon waves, 39 displayed t-wave inversion in the right precordial leads from V1 to V3, 28 in the left precordial leads from V4 to V6, and 11 in all precordial leads (from V1 to V6). EAM of the RV was performed on 54 (65.8%) patients. The bipolar EAM of the RV was pathologic for 30/54 (55.5%) patients, 38/54 (70.4%) in case of unipolar EAM, which was indicative of epicardial dysfunction [22,23,24]. EAM of the LV was performed on 25 (30.5%) patients. Bipolar EAM of the LV resulted pathologic for 14/25 (56%) patients, 17/25 (68%) in case of unipolar EAM.

In the total population, 61 (74.4%) subjects were prescribed antiarrhythmic therapy, including class 1C antiarrhythmics, amiodarone and beta-blockers, and 29 (35.4%) patients with ACE-inhibitors or angiotensin receptor blockers. For 24 (29.3%) cases, the clinicians deemed the trans-catheter ablation procedure necessary to treat ventricular arrhythmias, and for 52 (63.4%) cases, the ICD implantation was necessary for primary or secondary prevention. Right, left or both ventricular biopsies were collected in 55 (67.1%) patients for diagnostic aims and samples from 20/55 (36.4%) patients were pathologic for ACM, 16/55 (29.1%) also displayed inflammatory infiltrates, while in 19 patients, the histological analysis provided non-conclusive findings.

Cardiac imaging examinations revealed the presence of areas of late gadolinium enhancement, indicative of fibrotic myocardial replacement, in 46/71 (64.8%), cardiac adipose infiltration in 41/71 (57.7%) and both fibrosis and fat in 32/71 (45.1%) probands. From CMR, it emerged that most patients, 28/71 (39.4%), showed biventricular dysfunction, whereas only 18/71 (25.5%) and 13/71 (18.3%) exhibited an exclusively right or left ventricular substrate disease, respectively.

### 3.2. Rare genetic Variants

Genetic screening of the 82 patients identified 3526 variants. We restricted the analysis to the 2803 high-quality variants with a read coverage above 30× only. Among these, the 283 variants with an allele frequency below the threshold (6.7 × 10^−5^) were defined as rare (Supplementary Figure S1) [20].

#### 3.2.1. Rare Genetic Variants in ACM Genes

**Figure 1:**
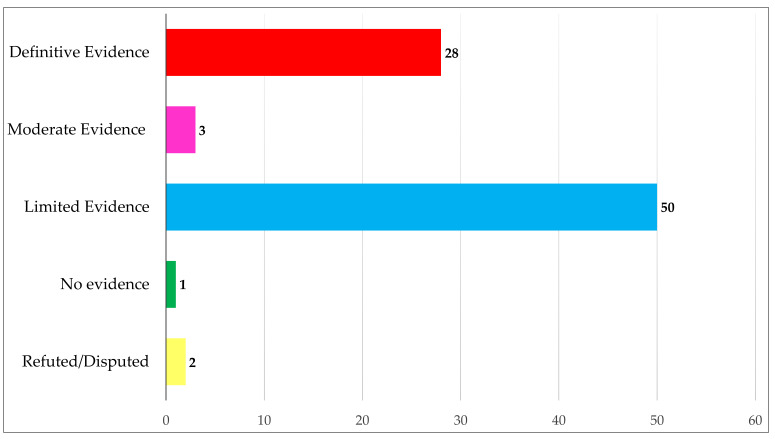
Classification of rare variants according to the association of the relative gene with ACM: definitive evidence (*PKP2*, *DSP*, *DSG2*, *DSC2*, *JUP*, and *TMEM43*), with moderate evidence (*DES* and *PLN*), with limited evidence (*SCN5A*, *LMNA*, *CDH2*, *CTNNA3*, *TGFB3*, *TTN*, *TJP1*, *MYH7*, *MYBPC3* and *MYL3),* with no evidence (*TNNC1*, *TNNI3*, *TNNT2*, *TPM1*, *ACTC1* and *MYL2*) and refuted/disputed (*RYR2* and *LDB3*) [7].

Among the rare variants, 81 (29%) were found in genes that are associated with ACM with definitive, moderate and limited evidence, as reported by the Arrhythmogenic Right Ventricular Cardiomyopathy Gene Curation Expert Panel [7] (Figure 1).

The selected 81 variants identified in ACM-associated genes were classified, according to ACMG/AMP guidelines [21]: 15 variants were classified as pathogenic or likely pathogenic, while 66 were classified as variant of uncertain significant (VUS) (Supplementary Table S2; Supplementary Figure S1).

**Figure 2:**
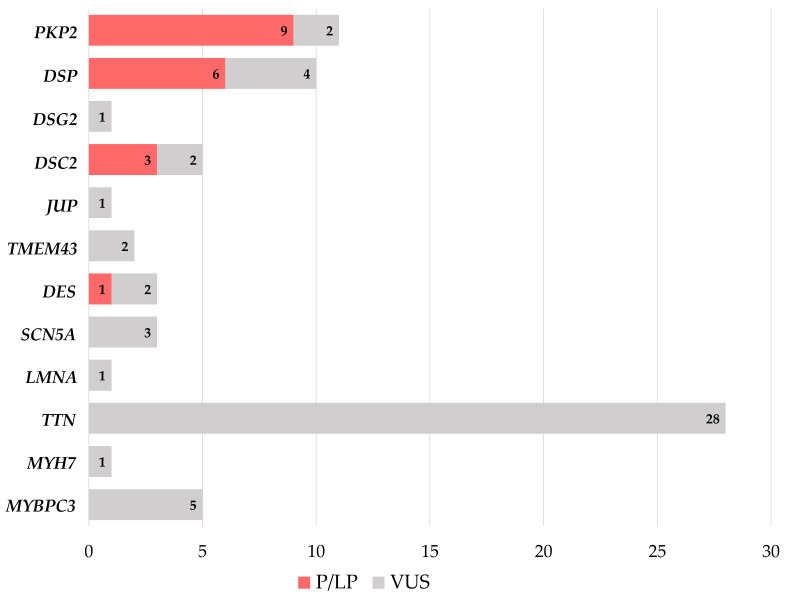
Distribution of carriers of rare variants in genes associated with ACM (with definite, moderate and limited evidence), identified in our cohort. Abbreviations: P: pathogenic; LP: likely pathogenic; VUS: variant of uncertain significance.

Regarding the 82 studied probands, we found that the 15 (likely) pathogenic variants were distributed in 19 (23%) carriers (Figure 2): 9 probands carried a variant in *PKP2*, 6 in *DSP*, 3 in *DSC2*, while 1 patient carried a pathogenic variant in *DES* (Figure 2). Of note, the variant in *PKP2* c.2013delC was found in four different probands, and the variant in *PKP2* c.1643delG was found in two others. The 66 VUS in ACM-associated genes were distributed in 40 patients: 8 (10%) probands carried both (likely) pathogenic variants and VUS, while 32 (39%) carried only VUS (Figure 2). We found that 2 probands carried VUS in *PKP2*, 4 in *DSP*, 1 in *DSG2*, 2 in *DSC2*, 1 in *JUP*, 2 in *TMEM43*, 2 in *DES*, 3 in *SCN5A*, 1 in *LMNA*, 28 in *TTN*, 1 in *MYH7* and 3 in *MYBPC3* (Figure 2).

Results of the genetic screening for each patient are reported in Supplementary Table S2.

#### 3.2.2. Rare Genetic Variants in Non-ACM Genes

In total, 202 variants were identified in genes associated with inherited cardiovascular disease but not with ACM.

Of these, 2 variants were in a gene recently reclassified as disputed (*RYR2*) and one was in a gene without clear evidence of association with ACM (*TNNT2*; Figure 1) [7]. 

Due to the lack of association with ACM, all the 202 variants were classified as VUS. However, 4 variants were ranked as radical for their high impact on the coded protein (Table 2; Supplementary Figure S1; Supplementary Table S2). The carriers of the variants in *DPP6* and in *ABCC9* were also carriers of pathogenic mutations in *PKP2*.

### 3.3. Genotype-Phenotype Correlation between Common Genetic Variants and Patient Characteristics

We identified 2520 common variants in the analyzed 174 genes (allele frequency ≥ 6.7 × 10^−5^). All the 82 probands carried at least one common variant. For the correlation analysis, we filtered away variants whose minor allele was present in less than 5 patients. The remaining 1320 had the potential to be the most informative. The genotype-phenotype correlation analysis involved 69 clinical variables, of which 32 were dichotomous categorical and 36 were continuous.

**Figure 3:**
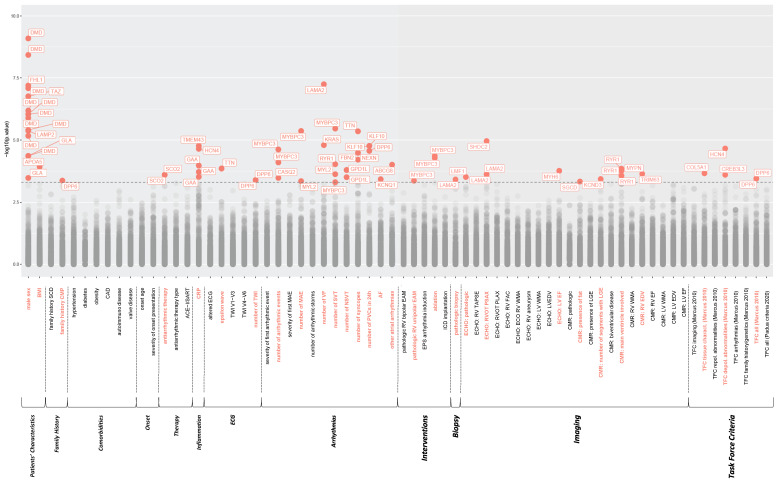
Manhattan plots representing the genotype-phenotype correlation. The 69 significant associations (*p* < 0.005) are shown in the graphs: 62 different genetic variables distributed over 29 (in red) of the 69 clinical parameters considered. For simplicity, variants are indicated by the name of the gene in which they have been identified. For polymorphism details, see Tables S3 and S4. “MAE” included SVT, syncope and VF. Abbreviations: BMI: body mass index; EPS: electrophysiological study; ECHO: echocardiogram; TAPSE: tricuspid annular plane systolic excursion; RVOT: right ventricular outflow tract; PSAX: parasternal short-axis; PLAX: parasternal long-axis; FAC: fractional area change; LV: left ventricle; EDV: end-diastolic volume; EF: ejection fraction; TCF: task force criteria.

We found that 62 different common genetic variants were significantly related to 29 different clinical parameters. All the 62 variants had an allele frequency higher than 1%. Of note, 5 variants were associated with more than one clinical variable, and 16 clinical variables were associated with more than one variant for a total of 69 associations. Among the 69 total hits, represented in Figure 3, 31 involved dichotomous categorical parameters revealing an association with genetic variants (Supplementary Table S3), whereas the remaining 38 continuous variables showed a linear correlation between genotype and phenotype (Supplementary Table S4).

We identified 28 associations (40.58%) involving primary or secondary arrhythmic phenotypes, such as the number of major arrhythmic events (MAE) or antiarrhythmic drug prescriptions, respectively. Most of these variants were located in genes associated with inherited arrhythmias and arrhythmic cardiomyopathies, such as *MYBPC3*, *KCNQ1*, *TTN*, *CASQ2*, *GPD1L*, *DPP6*, *HCN4* and *NEXN*. In particular, 3 variants resulted linked with multiple arrhythmic phenotypes: *MYBPC3*:c.3288G>A, *MYBPC3*:c.2308+18C>G and *MYL2*:c.132T>C.

Interestingly, *KCNQ1*:c.1394-39T>G and *HCN4*:c.1979-41A>G variants were found linked with atrial fibrillation in the patient and the fulfillment of depolarization abnormalities diagnostic criteria, respectively.

Eight (11.59%) hits concerned substrate defects, such as fibro-adipose substitution or RV end-diastolic volume (EDV), and they mainly involved genes associated with cardiomyopathies and muscle dysfunction, including *TRIM63*, *MYH6*, *SGCD* and *LAMA2*. Similarly, the gene variant *COL5A1*: c.2799+22C>T was correlated to the achievement of diagnostic criteria regarding the characterization of the ventricular walls; and 3 different polymorphisms located on the *RYR1* gene (c.7835+5A>G; c.8693-10G>C; c.8068-29_8068-27del) were associated with cardiomyopathy with prevalent LV involvement.

Five associations (7.25%) were found with increased levels of the marker of inflammation CRP.

Notably, as a confirmation of the effectiveness of the analysis, we found that all the 15 variants associated with the male sex phenotype were localized on the X chromosome. Similarly, the polymorphism c.457G>A on *APOA5* correlated with higher body mass index.

### 3.4. An Increased Arrhythmic Risk Is Associated with Selected Common Genetic Variants

Four previously identified polymorphisms (*MYBPC3*:c.3288G>A, *MYL2*:c.132T>C, *MYBPC3*:c.2308+18C>G and *CASQ2*:c.1194T>C), associated with the occurrence of arrhythmias, were further used for survival analysis to assess the predisposition of the different genotypes to the occurrence of major and/or minor arrhythmic events in the follow-up [25].

**Figure 4:**
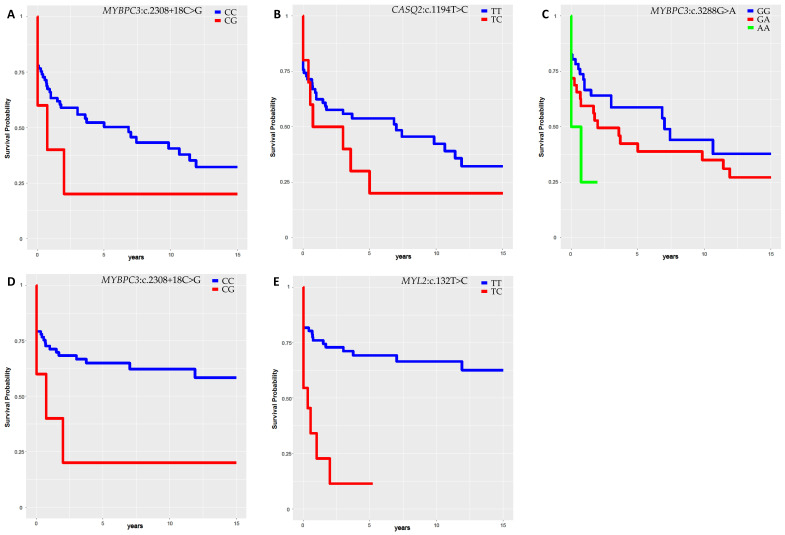
Kaplan–Meier curves representing the arrhythmic events-free survival during follow-up time in association with *MYBPC3*:c.2308+18C>G variants (**A**), *CASQ2*:c.1194T>C (**B**) and *MYBPC3*:c.3288G>A (**C**). Kaplan-Meier curves representing the MAE-free survival during follow-up time in association with *MYBPC3*:c.2308+18C>G (**D**) and *MYL2*:c.132T>C (**E**) variants. “MAE” includes syncope, sustained ventricular tachycardia and ventricular fibrillation. “Arrhythmic event” includes NSVT, syncope, SVT and VF.

Results showed that patients carrying the variants *MYBPC3*:c.3288G>A (32/82 heterozygotes and 4/82 homozygotes), *MYBPC3*:c.2308+18C>G (5/82 heterozygotes) and *CASQ2*:c.1194T>C (10/82 heterozygotes and 2/82 homozygotes) displayed increased susceptibility to develop arrhythmias (MAE+NSVT) in the follow-up (Figure 4A–C) (*MYBPC3*:c.3288G>A WT vs. heterozygous: HR = 2.45 (0.92–6.53), *p* = 0.07; homozygous vs. heterozygous: HR = 3.69 (0.02–4.76), *p* = 0.46; homozygous vs. WT: HR = 2.74 (0.92–8.17), *p* = 0.06; *MYBPC3*:c.2308+18C>G HR = 3.12 (0.4–24.3), *p* = 0.27; *CASQ2*:c.1194T>C HR = 2.25 (0.44–10.36), *p* = 0.29).

Likewise, the carriers of the polymorphisms c.2308+18C>G in *MYBPC3* (5/82) and c.132T>C in *MYL2* (11/82) exhibited a higher risk of manifesting MAE (respectively, HR = 7.48 (0.92–61.1), *p* = 0.06 and HR = 20.14 (4.1–99.6), *p* = 0.0002; Figure 4D,E).

Since carriers of common variants can also carry ACM pathogenic or likely pathogenic variants, we examined the influence of the latter on the arrhythmia susceptibility. As shown in Supplementary Figure S3, no significant influence was detected.

## 4. Discussion

In the present study, we analyzed the genetic data of a consecutive series of ACM patients, examining both rare and common genetic variants. Our goals were to describe the genetic characteristics of our population evaluating the pathogenicity or the impact of rare variants and to evaluate the potential impact of common variants on the ACM phenotype.

The frequency of pathogenic/likely pathogenic variants in ACM-associated genes of our cohort is relatively low (23%) compared with other described ACM populations. However, the adjudication approaches are really variable among different cohorts [6,26,27,28], and we used a stringent variant classification based on ACMG/AMP guidelines.

Interestingly, we did not find pathogenic variants in the genes classified as having limited or no evidence of association with ACM nor in the disputed genes.

A consistent number of VUS was identified in the ACM-associated genes. Specific functional validations and segregation analysis within the family could help to understand whether these variants can be reclassified as (likely) pathogenic. In addition, our report represents a benchmark for addressing their frequency in other ACM cohorts.

Several observations can be made about rare variants in ACM-associated genes.

Different patients carried either a pathogenic mutation and a VUS or two VUS on the same gene. Depending on the *cis* or *trans* allele localization of the two variants, we can have different interpretations. For instance, patient s86 carried two variants in *DES*: a VUS (c.266T>C) and a pathogenic frameshift (c.268_269insC). If the two variants were on the same allele, a lower impact of the missense compared to the frameshift mutation is expected. However, we cannot exclude their localization on two different alleles and a compound heterozygous effect: therefore, the patient might not be able to produce any fully functioning DES proteins. The association of *DES* mutation with ACM phenotype has moderate evidence according to the Arrhythmogenic Right Ventricular Cardiomyopathy Gene Curation Expert Panel [7], whereas it is known to be linked to dilated cardiomyopathy (DCM) and left ventricular non-compaction (LVNC) [29,30,31]. Nevertheless, our patient had a typical biventricular form of ACM with mainly right involvement and only a mild LV dilation, without trabeculation; thus, clinical features that did not overlap with DCM or LVNC.

Another interesting finding is the homozygous frameshift mutation in *DSC2* (c.2398_2399insG) found in patient s20. Recessive desmosomal mutations are usually associated with cardiocutaneous syndromes presenting clinical and histopathological features of ACM [8]. In addition to Naxos and Carvajal diseases, homozygous mutations of *DSC2* cause ACM with mild palmoplantar keratoderma and woolly hair [32]. Our proband did not display any cardiocutaneous phenotype. In accordance with the literature, describing the occurrence of homozygous *DSC2* pathogenic variants in patients with predominantly biventricular ACM [33], patient s20 displayed a biventricular disease.

Among rare variants, we identified 4 high-impact variants in genes associated with cardiovascular inherited diseases but never specifically to ACM. The selection of these variants was based on a high-impact consequence on the protein frame, length, and splicing in genes where the loss of function is a known disease mechanism. These variants may be incidental findings linked to other cardiovascular conditions. Alternatively, they may have a to date unknown relevance for ACM, either as a primary cause of the disease or as a phenotypic modulator. We accurately analyzed the clinical state of every single patient carrying these variants to evaluate a potential phenotypic overlapping with the other pathologies to which the mutated genes are associated.

Patient s07, carrier of the variant *APOB*:c.7537C>T, exhibited a definite biventricular ACM form, with mainly RV involvement. Variants in *APOB* are associated with hypercholesterolemia and ischemic risk [34,35]. s07 did not display hypercholesterolemia, but myocardial infarction occurred in two family members (the father and a paternal uncle) of our proband. Nevertheless, clinical suspicion of ACM was formulated for both. In addition, another paternal uncle died of SCD, without previous signs of infarction, and a paternal cousin showed ventricular tachycardia and syncope (the pedigree of the family is reported in Supplementary Figure S2). Genetic analysis in the father and the cousin of the proband would clarify the segregation of the *APOB* variant with ACM suspicion within this family. In case an ACM causative role of the variant was excluded, this variant could either be considered an incidental finding [36], or we can speculate about a function as a rare phenotype modulator. In fact, *APOB* encodes the major constituent protein of low-density lipoproteins [37], which, in an oxidized state, play a modulatory role in ACM adipogenesis [38].

Patient s81 carried two variants: *PKP2*:c.1378+1G>C and *ABCC9*:c.284+1G>A. We assumed that the likely pathogenic variant in the *PKP2* gene was responsible for the disease. Defects in *ABCC9* have been identified in DCM and AF patients. *ABCC9* variants impair the ATP-sensitive potassium channel function, and through this mechanism, it is believed to facilitate arrhythmogenesis in DCM and atrial fibrillation (AF) [39,40]. Thus, we can speculate on a potential contribution of *ABCC9* mutations to ACM arrhythmic phenotype in our s81 patient, which indeed showed several events of SVT.

Variants in *MIB1* are associated with LVNC, but patient s31, carrying the variant *MIB1*:c.376C>T, displayed a typical LV form of ACM, with extensive fibro-adipose substitution LV free wall and enlarged LV without trabeculation [41,42].

Proband s14 carried the splicing variant c.2078+5G>A in *DPP6*, which is a gene that is reported to be associated with idiopathic ventricular fibrillation [43,44]. Our patient carried also the pathogenic variants *PKP2*:c.2013delC, which is likely the causative ACM variant; nevertheless, we cannot exclude a possible impact of the variant in *DPP6* on the phenotype.

Therefore, we can speculate that the above-mentioned genes, found mutated in our ACM patients, could be considered candidates for validation as new ACM-associated genes. Functional studies on the specific variants are awaited to confirm the pathogenic causative or contributory role in ACM [45,46].

As concerns common variants, a genotype-phenotype correlation was performed. Although it is accepted that polymorphisms might contribute to disease variable expressivity, this is the first time that a similar analysis has been performed in ACM patients on all the genes of the cardiovascular TruSight™ Cardio Sequencing panel. Indeed, to our knowledge, the search for frequent disease modifier has been achieved only for variants in the *RYR2* gene [19].

Among variants directly associated with arrhythmic events or indirectly with antiarrhythmic therapies/interventions, we recognized variants in genes involved in arrhythmic disease (e.g., *MYBPC3, KCNQ1, HCN4, TTN, CASQ2, GPD1L, DPP6,* and *NEXN*). Of note, the polymorphisms c.1394-39T>G in *KCNQ1*, one of the main genes associated with familial atrial fibrillation [47], was found linked to the presence of atrial fibrillation in ACM patients. Likewise, the variant c.1979-41A>G in *HCN4*, the gene coding for the main channel responsible for pacemaker current and linked to different rhythm disorders, such as bradycardia, resulted related to depolarization abnormalities in patients [48,49]. For other variants, whose gene are not directly involved in arrhythmias, an indirect effect could be postulated. For example, the three variants linked to multiple variables in the arrhythmia category (*MYBPC3*:c.3288G>A, *MYBPC3*:c.2308+18C>G and *MYL2*:c.132T>C) are in genes associated with hypertrophic cardiomyopathy (HCM), where arrhythmias are secondary to ventricular remodeling [50,51]. Interestingly, the survival analysis of 4 variants confirmed the association of the minor allele with the increased risk of arrhythmic events during the course of the disease.

Among associations concerning substrate defects, we found polymorphisms in genes related to muscle dysfunctions and cardiomyopathies. For instance, the variant *MYH6*:c.5164-22A>G correlated with the reduction in LV ejection fraction in ACM patients. Previous studies described how *MYH6* defects contribute to ventricular remodeling, causing a spectrum of phenotypes comprising DCM, HCM [52], ischemic cardiomyopathy and HF [53]. It is noteworthy that the *TRIM63*:c.267G>T variant correlated with increased RV EDV. Muscle Ring Finger 1 (MURF1), encoded by the *TRIM63* gene, maintains sarcomere proteins homeostasis, whose impairments have been reported to cause HCM [54,55]. The variant *COL5A1*:c.2799+22C>T was correlated with the fulfillment of TCF concerning the tissue characterization of the ventricular walls: indeed, *COL5A1* encodes collagen type V α1 chain, which is a constituent of heart scars that regulates scar size in an integrin-dependent manner [56]. Defects in *COL5A1* are associated with Ehlers-Danlos syndrome, which can present cardiac involvement, although this is uncommon [57,58].

Unexpected but relevant findings were three polymorphisms in *RYR1* associated with prevalent LV involvement in ACM. A previous work reported an association between *RYR1* polymorphisms and LV hypertrophy, declaring the need for further examination through functional analysis of *RYR1* to understand the underlying mechanism [59].

Several limitations have to be acknowledged for the present study. (i) We analyzed, by NGS, only a subset of the genes possibly linked to cardiovascular diseases (those included in the TruSight™ Cardio Sequencing panel). However, other genes (e.g., *FLNC*) have recently been reported as relevant for genetic cardiovascular conditions [60]. In addition, 3 genes with limited evidence of association with ACM are not in our panel (*CDH2*, *CTNNA3* and *TJP1*). A more comprehensive analysis, such as exome or genome sequencing, could overcome this limitation, including all the coding genes or/and intronic regulatory regions. In a third instance, specific assays, such as karyotype, MLPA or CGH arrays could be run in parallel with NGS, to detect also big genome rearrangements and copy number variations. (ii) The identified high-impact rare variants would need to be screened in other ACM patients from international large cohorts to establish a possible association with ACM. (iii) Segregation studies would also help confirm causality. (iv) Regarding the genotype-phenotype correlation, the relatively limited sample size allowed only a preliminary association between variants and phenotypes, which will need confirmation in larger cohorts. (v) Furthermore, we cannot exclude that some loci, important for certain phenotypes, may be in linkage disequilibrium with the identified variant.

## 5. Conclusions

In conclusion, we provide a complete molecular diagnostic workout on a single-center cohort of ACM patients, leading to the identification of causative mutations in ACM genes and the report of different VUS, to be considered for future evaluation. In addition, by broadening the panel of analyzed genes to 174 genes linked to cardiovascular genetic diseases, we found 4 high-impact variants, which can potentially be taken into account as ACM causative or modifier candidates. In addition, few frequent variants may act as phenotypic modifiers. These findings pave the way for novel and deep genetic studies to overcome the present genetic limitations concerning ACM.

## Figures and Tables

**Table 1:** Summary of the main clinical characteristics of the ACM patient cohort, expressed as the number of patients over the total and as a percentage. Abbreviations: SCD: sudden cardiac death; CMP: cardiomyopathy; CAD: coronary artery disease; ACE-I: angiotensin-converting enzyme inhibitors; ARB: angiotensin receptor blockers; ICD: implantable cardioverter-defibrillators; NSVT: non-sustained ventricular tachycardia; SVT: sustained ventricular tachycardia; VF: ventricular fibrillation; ECG: electrocardiogram; PVCs: premature ventricular contractions; TWI: T wave inversion; RV: right ventricle; EAM: electro-anatomical mapping; LV: left ventricle; CMR: cardiac magnetic resonance. “MAE” included SVT, syncope and VF.

Categories	Parameters	n	%
**Sex**	male sex	66/82	80.5%
**Family history**	family history ACM	8/82	9.8%
	family history SCD	12/82	14.6%
	family history CMP	17/82	20.7%
**Lifestyle**	smoke	16/82	19.5%
	sport	31/82	37.8%
	sport endurance	9/82	11.0%
**Comorbidities**	hypertension	17/82	20.7%
	diabetes	2/82	2.4%
	obesity	4/82	4.9%
	CAD	8/82	9.8%
	autoimmune disease	8/82	9.8%
**Biopsy**	ACM features	20/55	36.4%
	ACM + inflammatory infiltrates	16/55	29.1%
**Therapy**	ACE-I/ARB	29/82	35.4%
	β-blockers	34/82	41.5%
	1C antiarrhythmics	5/82	6.1%
	amiodarone	22/82	26.8%
**Medical interventions**	trans-catheter ablation	24/82	29.3%
	ICD implant	52/82	63.4%
**Arrhythmias**	MAE	35/82	42.7%
	NSVT + MAE	50/82	61.0%
	PVCs	39/82	47.6%
	atrial arrhythmias	15/82	18.3%
	events in the follow-up (NSVT + MAE)	21/82	25.6%
**ECG**	ECG alterations	64/82	78.0%
	epsilon wave	4/82	4.9%
	TWI V1–V3	39/82	47.6%
	TWI V4–V6	28/82	34.1%
	TWI V1–V6	11/82	13.4%
**EAM**	pathologic RV bipolar EAM	30/54	55.5%
	pathologic RV unipolar EAM	38/54	70.4%
	pathologic LV bipolar EAM	14/25	56%
	pathologic LV unipolar EAM	17/25	68%
**CMR**	exclusively RV disease	18/71	25.5%
	exclusively LV disease	13/71	18.3%
	biventricular disease	28/71	39.4%%
	exclusively fibrosis	11/71	15.5%
	exclusively adipose infiltration	6/71	8.5%
	fibrosis + adipose infiltration	32/71	45.1%

**Table 2:** Schematic summary of the 4 rare radical genetic variants in genes associated with cardiovascular diseases but not with ACM. Abbreviations: LVNC: left ventricular non-compaction; n.a.: not applicable; DCM: dilated cardiomyopathy; AF: atrial fibrillation.

Gene	Patient	Consequence	Genome Position	CDS Position	Protein Position	rs ID	Main Known Gene-Associated Diseases
*APOB*	s07	stop gained	chr2:21232203	c.7537C>T	p.R2513*	rs146538280	hypercholesterolemia/cardiac ischemia
*MIB1*	s31	stop gained	chr18:19345879	c.376C>T	p.R126*	rs190657514	LVNC
*DPP6*	s14	splicing	chr7:154667815	c.2078+5G>A	n.a.		idiopathic ventricularfibrillation
*ABCC9*	s81	splicing	chr12:22086715	c.284+1G>A	n.a.	rs762907980	DCM/AF

## Data Availability

Not applicable.

## Supplementary Materials

The following supporting information can be downloaded at: https://www.mdpi.com/article/10.3390/biom12081043/s1, Table S1: TruSight™ Cardio Sequencing list of 174 genes reported being associated with 15 inherited cardiac conditions; Table S2: Schematic summary of the 141 rare genetic variants classified as variants of uncertain significance (VUS), pathogenic (P) or likely pathogenic (LP); Table S3: Contingency table classifying the number of patients carrying the variants for each dichotomous categorical variables for the 31 significant (*p* ≤ 0.005) associations; Table S4: Summary of 38 significant (*p* ≤ 0.005) linear correlations between continuous variables and common genetic variants; Figure S1: Analysis workflow for filtering and selection of rare and common variants; Figure S2: Family pedigree of patient s07; Figure S3: Kaplan Meier curves representing the arrhythmic events-free survival during follow-up time in coexistence with ACM pathogenic/likely pathogenic variants.

## Abbreviations

**ACM**	arrhythmogenic cardiomyopathy
**ACMG/AMP**	American College of Medical Genetics and Genomics/Association for Molecular Pathology
**AF**	atrial fibrillation
**CMR**	cardiac magnetic resonance
**DCM**	dilated cardiomyopathy
**DES**	desmin
**DSC2**	desmocollin-2
**DSG2**	desmoglein-2
**DSP**	desmoplakin
**EAM**	electro-anatomical mapping
**ECG**	electrocardiogram
**ECHO**	echocardiography
**EDV**	end-diastolic volume
**EPS**	electrophysiological study
**HCM**	hypertrophic cardiomyopathy
**HF**	heart failure
**ICD**	implantable cardioverter defibrillator
**JUP**	plakoglobin
**LV**	left ventricle
**LVNC**	left ventricular noncompaction cardiomyopathy
**MAE**	major arrhythmic event
**NGS**	next generation sequencing
**NSVT**	non-sustained ventricular tachycardia
**PKP2**	plakophilin-2
**PLN**	phospholamban
**PVC**	premature ventricular contraction
**RV**	right ventricle
**SCD**	sudden cardiac death
**SVT**	sustained ventricular tachycardia
**TCF**	task force criteria
**TMEM43**	transmembrane protein 43
**VF**	ventricular fibrillation
**VUS**	variant of uncertain significance
