# Supplementary material for: Spectrum of Rare and Common Genetic Variants in Arrhythmogenic Cardiomyopathy Patients
Source: Biomolecules. 2022 Jul 28;12(8):1043. doi: 10.3390/biom12081043 (PMC9405889; doi:10.3390/biom12081043)
Supplement: Supplementary file 1 [file biomolecules-12-01043-s001.zip › biomolecules-1806599-supplementary-proof done-update.pdf]

biomolecules MDPI

Supplementary Materials

Supplementary Table S1. TruSight™ Cardio Sequencing list of 174 genes reported being associated with 15 inherited cardiac conditions (aortic valve disease, Marfan syndrome, Loeys-Dietz syndrome, short QT syndrome, catecholaminergic polymorphic ventricular tachycardia, familial hypercholesterolemia, restrictive cardiomyopathy, non-compaction cardiomyopathy, Noonan syndrome, arrhythmogenic right ventricular cardiomyopathy, Brugada syndrome, structural heart disease, long QT syndrome, familial aortic aneurysm, familial atrial fibrillation, hypertrophic cardiomyopathy, dilated cardiomyopathy).

**TruSight™ Cardio Sequencing gene panel list**								
ABCC9	*CACNB2*	*DOLK*	*GJA5*	*KCNJ5*	*MYH11*	*PRDM16*	*SGCG*	*TNNC1*
ABCG5	*CALM1*	*DPP6*	*GLA*	*KCNJ8*	*MYH6*	*PRKAG2*	*SHOC2*	*TNNI3*
ABCG8	*CALR3*	*DSC2*	*GPD1L*	*KCNQ1*	*MYH7*	*PRKAR1A*	*SLC25A4*	*TNNT2*
ACTA1	*CASQ2*	*DSG2*	*GPIHBP1*	*KLF10*	*MYL2*	*PTPN11*	*SLC2A10*	*TPM1*
ACTA2	*CAV3*	*DSP*	*HADHA*	*KRAS*	*MYL3*	*RAF1*	*SMAD3*	*TRDN*
ACTC1	*CBL*	*DTNA*	*HCN4*	*LAMA2*	*MYLK*	*RANGRF*	*SMAD4*	*TRIM63*
ACTN2	*CBS*	*EFEMP2*	*HFE*	*LAMA4*	*MYLK2*	*RBM20*	*SNTA1*	*TRPM4*
AKAP9	*CETP*	*ELN*	*HRAS*	*LAMP2*	*MYO6*	*RYR1*	*SOS1*	*TTN*
ALMS1	*COL3A1*	*EMD*	*HSPB8*	*LDB3*	*MYOZ2*	*RYR2*	*SREBF2*	*TTR*
ANK2	*COL5A1*	*EYA4*	*ILK*	*LDLR*	*MYPN*	*SALL4*	*TAZ*	*TXNRD2*
ANKRD1	*COL5A2*	*FBN1*	*JAG1*	*LDLRAP1*	*NEXN*	*SCN1B*	*TBX20*	*VCL*
APOA4	*COX15*	*FBN2*	*JPH2*	*LMF1*	*NKX2-5*	*SCN2B*	*TBX3*	*ZBTB17*
APOA5	*CREB3L3*	*FHL1*	*JUP*	*LMNA*	*NODAL*	*SCN3B*	*TBX5*	*ZHX3*
APOB	*CRELD1*	*FHL2*	*KCNA5*	*LPL*	*NOTCH1*	*SCN4B*	*TCAP*	*ZIC3*
APOC2	*CRYAB*	*FKRP*	*KCND3*	*LTBP2*	*NPPA*	*SCN5A*	*TGFB2*	
APOE	*CSRP3*	*FKTN*	*KCNE1*	*MAP2K1*	*NRAS*	*SCO2*	*TGFB3*	
BAG3	*CTF1*	*FXN*	*KCNE2*	*MAP2K2*	*PCSK9*	*SDHA*	*TGFBR1*	
BRAF	*DES*	*GAA*	*KCNE3*	*MIB1*	*PDLIM3*	*SEPN1*	*TGFBR2*	
CACNA1C	*DMD*	*GATAD1*	*KCNH2*	*MURC*	*PKP2*	*SGCB*	*TMEM43*	
CACNA2D1	*DNAJC19*	*GCKR*	*KCNJ2*	*MYBPC3*	*PLN*	*SGCD*	*TMPO*	

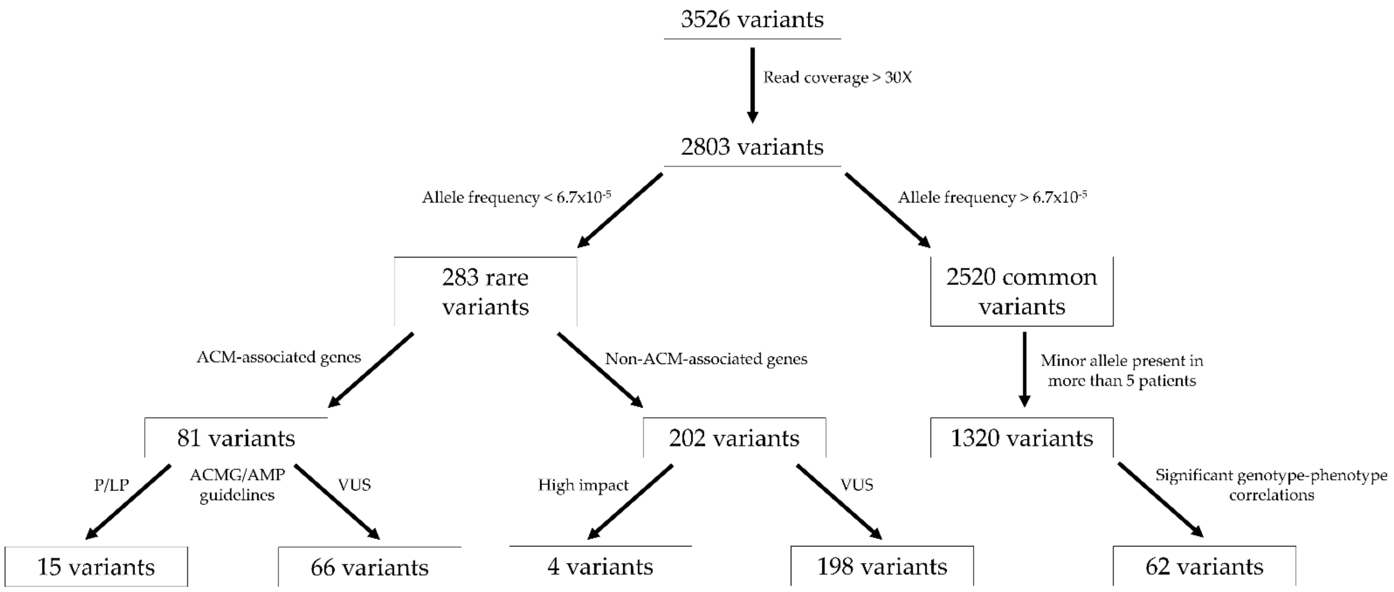

Supplementary Figure S1. Analysis workflow for filtering and selection of rare and common variants. Abbreviations: ACM: arrhythmogenic cardiomyopathy; P: pathogenic; LP: likely pathogenic; VUS: variant of uncertain significance; ACMG/AMP: American College of Medical Genetics and Genomics / Association for Molecular Pathology.

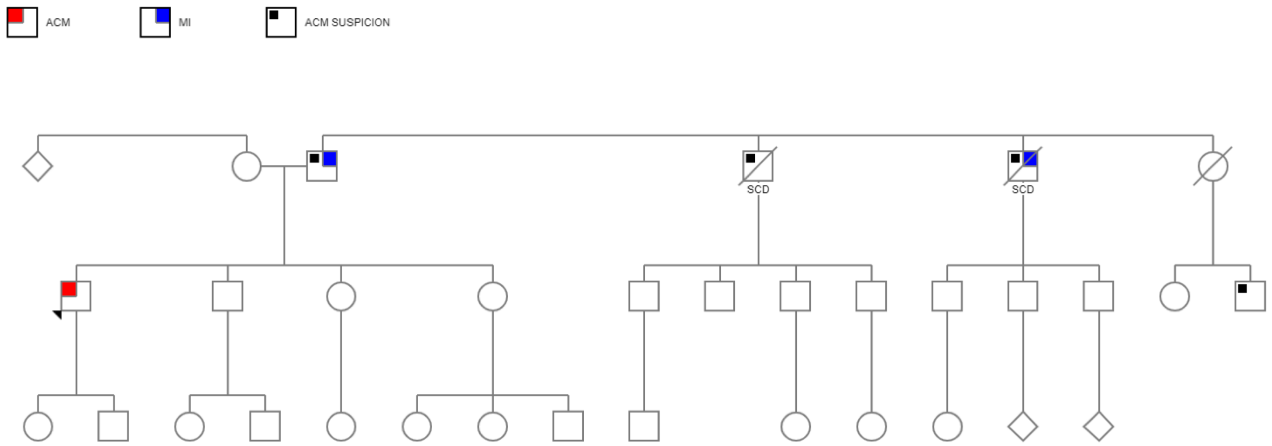

Supplementary Figure S2. Family pedigree of patient s07.

Supplementary Table S2. Schematic summary of the 141 rare genetic variants classified as VUS, LP or P. The carrier patient, the type of mutation, the position on the genome (taking hg19/GRCh37 genome as reference), the gene in which it is found, the nomenclature referring to the CDS and the protein, and the hetero- or homozygous status are indicated. The genes are color-coded according to their level of association with ACM: red=definitive evidence of association; pink=moderate evidence; blue=limited evidence; green=no evidence; yellow=refuted/disputed.

Patient	Stop gain	Frameshift	In-frame deletion	Splicing		Missense		
s15	Pathogenic *DSP* c.6850C>T p.R2284*							
s18		Pathogenic *PKP2* c.1643delG p.G548Vfs*15						
s83		Pathogenic *PKP2* c.1643delG p.G548Vfs*15						
s17		Pathogenic *PKP2* c.2013delC p.K672Rfs*12						
s77		Pathogenic *PKP2* c.2013delC p.K672Rfs*12						
s06		Pathogenic *PKP2* c.2013delC p.K672Rfs*12				VUS *DSG2* c.1088C>T p.S363L	VUS *TTN* c.38914A>G p.M12972V	
s14		Pathogenic *PKP2* c.2013delC p.K672Rfs*12		VUS *DPP6* c.2078+5G>A				
s03		Pathogenic *PKP2* c.1264_1265delTT p.L422Sfs*3				VUS *TTN* c.14425G>A p.G4809S		
s37		Pathogenic *PKP2* c.1238_1239insG p.A414Sfs*12				VUS *TTN* c.77296A>G p.I25766V		
s64		Pathogenic *DSP* c.4321_4322del p.Q1442Efs*8				VUS *TTN* c.60095A>T p.K20032M		
s44	Pathogenic *DSP* c.2000delG p.W667*					VUS *TTN* c.62434G>A p.G20812R		

s72	Pathogenic *DSP* c.1306C>T p.Q436*					VUS *TTN* c.55343T>C p.V18448A		
s75	Pathogenic *DSC2* c.268G>T p.E90*					VUS *DSP* c.7783A>G p.T2595A	VUS *TTN* c.16892T>C p.I5631T	VUS *TTN* c.2032A>G p.T678A
s81				Pathogenic *PKP2* c.1378+1G>C	VUS *ABCC9* c.284+1G>A			
s86		Pathogenic *DES* c.268_269ins C p.D90Afs*28				VUS *DES* c.266T>C p.L89P		
s20		Likely Patho-genic *DSC2* c.2398_2399in sG p.A800Gfs*37						
s25	Likely Patho-genic *DSP* c.3466C>T p.Q1156*							
s48	Likely Patho-genic *DSP* c.2821C>T p.R941*							
s70		Likely Patho-genic *DSC2* c.1234_1235in sT p.T412Ifs*3				VUS *PKP2* c.2006G>A p.S669N		
s01						VUS *TTN* c.66758G>A p.G22253D		
s02						VUS *MYBPC3* c.3472G>A p.V1158I	VUS *TTN* c.7738A>G p.I2580V	
s04						VUS *TTN* c.54952C>T p.R18318C		
s08						VUS *MYBPC3* c.2807C>T p.T936M		
s09						VUS *TTN* c.70130T>C p.V23377A		

s10						VUS *PKP2* c.548G>A p.S183N		
s13						VUS *TTN* c.36328G>T p.V12110L	VUS *TTN* c.28303G>A p.D9435N	
s19						VUS *MYH7* c.2974C>A p.L992M		
s22						VUS *TTN* c.90036C>G p.D30012E	VUSI *TTN* c.55567G>A p.V18523	
s23						VUS *JUP* c.1359G>T p.E453D	VUS *TTN* c.72884C>A p.T24295K	VUS *TTN* c.62447A>G p.D20816G
s26						VUS *TTN* c.77845G>T p.A25949S	VUS *TTN* c.62782C>T p.R20928W	VUS *TTN* c.38368C>T p.P12790S
s27			VUS *DSC2* c.2368_2370del p.G790del			VUS *DSC2* c.2497C>T p.R833C		
s28						VUS *DSP* c.860A>G p.N287S		
s29						VUS *TTN* c.27348C>G p.D9116E		
s30						VUS *TMEM43* c.1052T>C p.F351S	VUS *TTN* c.100639A>C p.I33547L	VUS *TTN* c.55391T>G p.V18464G
s41						VUS *TTN* c.69266G>A p.G23089D		
s42						VUS *TTN* c.30554C>T p.S10185L	VUS *SCN5A* c.1919C>T p.P640L	
s47						VUS *DSC2* c.416C>T p.P139L		

s52			VUS homo *MYBPC3* c.1102_1104del p.K368del			VUS *DSP* c.2019G>C p.Q673H		
s53						VUS *TTN* c.50861C>G p.T16954R	VUS *TTN* c.39569G>A p.G13190E	
s55						VUS *TTN* c.17296A>G p.G13190E	VUS *TTN* c.3589G>A p.V1197I	
s57						VUS *TTN* c.31274C>T p.S10425L		
s59						VUS *SCN5A* c.1901C>G p.S634W		
s60				VUS *TMEM43* c.705+7G>A		VUS *TTN* c.97313G>A p.R32438K	VUS *TTN* c.87176T>C p.F29059S	
s61						VUS *DSP* c.5642T>C p.I1881T		
s63						VUS *TTN* c.41246C>T p.P13749L	VUS *SCN5A* c.3107A>G p.Q1036R	
s64						VUS *TTN* c.60095A>T p.K20032M		
s69						VUS *TNNT2* c.616C>T p.R206W	VUS *MYBPC3* c.760C>T p.L254F	VUS *TTN* c.38526T>A p.D12842E
s74						VUS *RYR2* c.3013A>G p.N1005D	VUS *MYBPC3* c.67G>A p.A23T	
s76						VUS *TTN* c.22208T>C p.I7403T		
s78						VUS *RYR2* c.6295G>A p.V2099I	VUS *TTN* c.23971C>T p.P7991S	VUS *TTN* c.6727G>T p.D2243Y

s80						VUS *LMNA* c.497G>A p.R166Q	VUS *TTN* c.66029T>G p.I22010S	VUS *TTN* c.43123G>A p.A14375T
s86						VUS *DES* c.1238A>G p.E413G		
s07	VUS *APOB* c.7537C>T p.R2513*							
s31	VUS *MIB1* c.376C>T p.R126*							

Supplementary Table S3. Contingency table classifying the number of patients carrying the variants for each dichotomous categorical variable for the 31 significant ($p \leq 0.005$) associations. For each variant, we indicated the associated variable and the number of patients presenting the variable, clustered according to the three genotypes (wild type, heterozygous and homozygous). Abbreviations: CDS: coding sequence; WT: wild type; hetero: heterozygous; homo: homozygous; RV: right ventricle; EAM: electro-anatomical mapping; ECHO: echocardiogram; CMR: cardiac magnetic resonance; LV: left ventricle; AF: atrial fibrillation; CMP: cardiomyopathy.

Gene	rs ID	Genome position	CDS position	Variable	n WT patients presenting the variable	n hetero patients presenting the variable	n homo patients presenting the variable	*p*-value
SCO2	rs12148	chr22:50962208	c.633A>C	**antiarrhythmic therapy**	5/61	32/61	24/61	0.000249447
SCO2	rs140523	chr22:50962782	c.59G>C	**antiarrhythmic therapy**	5/61	32/61	24/61	0.000249447
MYBPC3	rs11570050	chr11:47371484	c.506-12del	**pathologic RV unipolar EAM**	1/38	22/38	15/38	0.000432308
LMF1	rs4984705	chr16:943107	c.664-35T>C	**ECO pathologic**	10/34	21/34	3/34	0.000306645
LAMA2	rs6569605	chr6:129807629	c.7760C>T	**pathologic biopsy**	2/36	20/36	14/36	0.000384499
MYBPC3	rs1052373	chr11:47354787	c.3288G>A	**ablation**	5/24	16/24	3/24	0.000043716
RYR1	rs1469695	chr19:38993372	c.7835+5A>G	**CMR main ventricle involved**	5/24	14/24	5/24	0.000269571
RYR1	.	chr19:38995355	c.8068-29_8068-27del	**CMR main ventricle involved**	5/24	14/24	5/24	0.000269571
RYR1	rs2915958	chr19:38997459	c.8693-10G>C	**CMR main ventricle involved**	6/24	13/24	5/24	0.000142771
MYBPC3	rs3729802	chr11:47354068	c.3627+49C>T	**ablation**	12/24	12/24	0/24	0.000053492
SGCD	rs1801193	chr5:155771579	c.84T>C	**CMR presence of fat**	16/41	9/41	16/41	0.000466120
MYPN	rs2200897	chr10:69908241	c.1245+17G>A	**CMR main ventricle involved**	15/24	8/24	1/24	0.000172586
KCNQ1	rs739502	chr11:2683152	c.1394-39T>G	**AF**	2/8	6/8	0/8	0.000373785
DPP6	rs2293353	chr7:154667628	c.1896A>G	**family history CMP**	11/17	5/17	1/17	0.000426630
ABCG8	rs4148217	chr2:44099433	c.1199C>A	**other atrial arrhythmias**	0/7	4/7	3/7	0.000098691
TTN	rs6715406	chr2:179650701	c.2244G>A	**epsilon wave**	0/4	0/4	4/4	0.000139767
GLA	rs2071228	chrX:100653109	c.1000-22C>T	**male sex**	54/66	0/66	12/66	0.000043550
GLA	rs2071397	chrX:100653950	c.640-16A>G	**male sex**	55/66	0/66	11/66	0.000332822
LAMP2	rs12097	chrX:119590533	c.156A>T	**male sex**	34/66	0/66	32/66	0.000004252
FHL1	rs2076705	chrX:135292022	c.889-8C>T	**male sex**	49/66	0/66	17/66	0.000000064
TAZ	rs62617809	chrX:153640406	c.-266G>A	**male sex**	51/66	0/66	15/66	0.000000173
DMD	rs2270672	chrX:31676096	c.8027+11C>T	**male sex**	46/66	0/66	20/66	0.000000650
DMD	rs1801188	chrX:31697636	c.7728T>C	**male sex**	51/66	0/66	15/66	0.000001285
DMD	rs1800275	chrX:31893307	c.-286+1A>C	**male sex**	52/66	0/66	14/66	0.000043888
DMD	rs3761604	chrX:31986430	c.6614+26G>T	**male sex**	45/66	0/66	21/66	0.000000082
DMD	rs1801187	chrX:32380996	c.5234G>A	**male sex**	35/66	0/66	31/66	0.000000004
DMD	rs228406	chrX:32503194	c.2645A>G	**male sex**	23/66	0/66	43/66	0.000000001
DMD	rs228373	chrX:32563263	c.2168+13T>C	**male sex**	44/66	0/66	22/66	0.000003962
DMD	rs115571	chrX:32563488	c.1993-37T>G	**male sex**	24/66	0/66	42/66	0.000006798
DMD	rs5927082	chrX:32591811	c.1704+51T>C	**male sex**	48/66	0/66	18/66	0.000000914
DMD	rs5927083	chrX:32591931	c.1635A>G	**male sex**	48/66	0/66	18/66	0.000000914

**Supplementary Table S4.**	**rs ID**	**Genome position**	**CDS position**	**Variable**	**R**	***p*-value**
SHOC2	rs11593866	chr10:112771339	c.1541-29A>T	**ECO RVOT PSAX**	0.6042512	0.00001106
LAMA2	rs55776770	chr6:129837320	c.9212-15C>A	**number of VF**	0.5623516	0.00000006
KCND3	rs640029	chr1:112321032	c.1518+26A>T	**CMR number of segments with LGE**	0.5479431	0.00036943
LAMA2	rs17057158	chr6:129670548	c.4523+19C>T	**ECO RVOT PSAX**	0.5213991	0.00024002
LAMA2	rs17057184	chr6:129691132	c.4956C>G	**ECO RVOT PSAX**	0.5213991	0.00024002
MYBPC3	rs3729948	chr11:47360053	c.2308+18C>G	**number of SVT**	0.4926425	0.00000345
TTN	rs72648907	chr2:179612383	c.14744G>A	**number of syncopes**	0.4877090	0.00000446
MYBPC3	rs3729948	chr11:47360053	c.2308+18C>G	**number of MAE**	0.4829326	0.00000432
TRIM63	rs35123100	chr1:26392824	c.267G>T	**CMR RV EDV**	0.4811918	0.00023016
TMEM43	rs34099410	chr3:14172381	c.222C>T	**CRP**	0.4800097	0.00001729
KLF10	.	chr8:103662353	c.*7G>A	**number of PVCs in 24h**	0.4742405	0.00001723
KLF10	.	chr8:103663390	c.1170G>A	**number of PVCs in 24h**	0.4742405	0.00001723
HCN4	rs12909882	chr15:73621946	c.1558C>T	**CRP**	0.4740870	0.00002267
KRAS	rs1137282	chr12:25362777	c.519T>C	**number of VF**	0.4620096	0.00001603
MYBPC3	rs3729948	chr11:47360053	c.2308+18C>G	**number of arrhythmic events**	0.4482575	0.00002409
NEXN	rs41312654	chr1:78399212	c.1251+48T>C	**number of syncopes**	0.4323942	0.00006192
RYR1	.	chr19:38980666	c.5815-50C>T	**number of SVT**	0.4225643	0.00009438
APOA5	rs3135507	chr11:116661488	c.457G>A	**BMI**	0.4222252	0.00011788
MYBPC3	rs1052373	chr11:47354787	c.3288G>A	**number of arrhythmic events**	0.4216149	0.00007985
MYH6	rs178637	chr14:23854272	c.5164-22A>G	**ECO LV EF**	0.4153594	0.00017241
GPD1L	rs2305361	chr3:32200322	c.619-46T>C	**number of NSVT**	0.4097513	0.00016040
MYL2	rs2301610	chr12:111353556	c.132T>C	**number of SVT**	0.4001450	0.00023545
COL5A1	rs76855839	chr9:137687183	c.2799+22C>T	**TFC tissue characterization**	0.3974486	0.00021788
CREB3L3	rs35474881	chr19:4154910	c.42C>T	**TFC depolarization abnormalities**	0.3940362	0.00024954
GPD1L	rs11351972	chr3:32188248	c.618+23del	**number of NSVT**	0.3930399	0.00031045
DPP6	rs35392762	chr7:154379517	c.785C>T	**number of TWI**	0.3883579	0.00040520
DPP6	rs75213895	chr7:154379739	c.1007C>T	**number of TWI**	0.3883579	0.00040520
CASQ2	rs28730711	chr1:116243868	c.1194T>C	**number of arrhythmic events**	0.3867453	0.00033183
DPP6	rs35392762	chr7:154379517	c.785C>T	**TFC all**	0.3849256	0.00035594
DPP6	rs75213895	chr7:154379739	c.1007C>T	**TFC all**	0.3849256	0.00035594
MYBPC3	rs1052373	chr11:47354787	c.3288G>A	**number of SVT**	0.3807412	0.00049387
MYL2	rs2301610	chr12:111353556	c.132T>C	**number of MAE**	0.3788648	0.00044826
GAA	rs2304836	chr17:78086846	c.2040+20A>G	**CRP**	-0.4112351	0.00030122
GAA	rs2304832	chr17:78090928	c.2331+20G>A	**CRP**	-0.4232299	0.00019102
GAA	rs1126690	chr17:78091405	c.2338G>A	**CRP**	-0.4379955	0.00010647
FBN2	rs190450	chr5:127614472	c.7200T>C	**number of syncopes**	-0.4467496	0.00003267
HCN4	rs481579	chr15:73616635	c.1979-41A>G	**TFC depolarization abnormalities**	-0.4500798	0.00002211
DPP6	rs3807218	chr7:154461112	c.723A>G	**number of PVCs in 24h**	-0.4641023	0.00002741

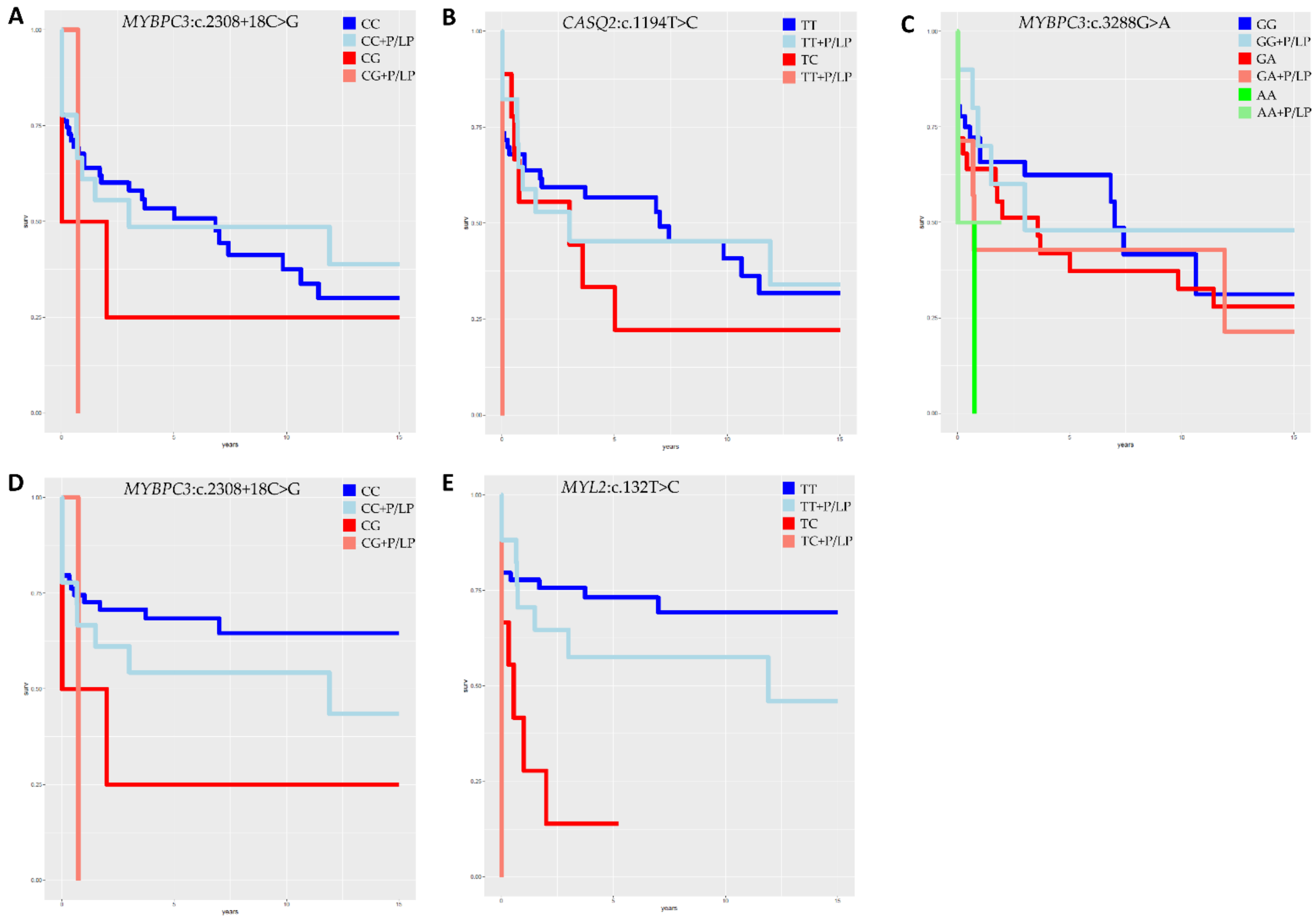

Supplementary Figure S3. Kaplan Meier curves representing the arrhythmic events-free survival during follow-up time in association with *MYBPC3*:c.2308+18C>G variants (**A**), *CASQ2*:c.1194T>C (**B**) and *MYBPC3*:c.3288G>A (**C)** in coexistence with ACM pathogenic/likely pathogenic variants. Kaplan Meier curves representing the MAE-free survival during follow-up time in association with *MYBPC3*:c.2308+18C>G (**D**) and *MYL2*:c.132T>C (**E**) variants, in coexistence with ACM pathogenic/likely pathogenic variants. The patients carrying pathogenic/likely pathogenic variants were indicated as "+P/LP". "MAE" includes syncope, sustained ventricular tachycardia and ventricular fibrillation. "Arrhythmic event" includes NSVT, syncope, SVT and VF.

Results showed that the presence of ACM pathogenic/likely pathogenic variants in patients carrying the polymorphisms *MYBPC3*:c.3288G>A, *MYBPC3*:c.2308+18C>G and *CASQ2*:c.1194T>C did not significantly influence the increased susceptibility to develop arrhythmias in the follow-up (S3A, B and C) (*MYBPC3*:c.3288G>A WT+P/LP vs WT: HR=0.89 [0.33–2.43], *p*=0.82; heterozygous+P/LP vs heterozygous: HR=1.08 [0.40–2.93], *p*=0.88; homozygous+P/LP vs homozygous: HR=0.4 [0.03–4.46], *p*=0.46. *MYBPC3*:c.2308+18C>G WT+P/LP vs WT: HR=0.93 [0.46–1.89], *p*=0.84; heterozygous+P/LP vs heterozygous: HR=1.65 [0.16–16.16], *p*=0.67. *CASQ2*:c.1194T>C WT+P/LP vs WT: HR=1.04 [0.51–2.15], *p*=0.91); heterozygous+P/LP vs heterozygous: HR=6.05 [0.71–51.35], *p*=0.1).

Likewise, the presence of pathogenic/likely pathogenic variants in patients carrying the variants *MYBPC3:*c.2308+18C>G and *MYL2:*c.132T>C did not significantly influence the higher risk of manifesting MAE (S3D and E). (*MYBPC3:*c.2308+18C>G WT+P/LP vs WT: HR=1.56 [0.70–3.45], *p*=0.27; heterozygous+P/LP vs heterozygous: HR=1.20 [0.12–11.7], *p*=0.87. *MYL2:*c.132T>C WT+P/LP vs WT: HR=1.64 [0.70–3.88], *p*=0.26; heterozygous+P/LP vs heterozygous: HR=3.1 [0.32–15.3], *p*=0.17).
